# A Case of Clonorchiasis in a Non-endemic Region of China

**DOI:** 10.7759/cureus.60725

**Published:** 2024-05-20

**Authors:** Jing Li, Qingbo Ren, Lina Wang, Peng Chen, Jinjin Li

**Affiliations:** 1 Clinical Laboratory, Qingdao Sixth People Hospital, Qingdao, CHN; 2 Central Laboratory, Qingdao Sixth People's Hospital, Qingdao, CHN; 3 Clinical Laboratory, Qingdao Sixth People’s Hospital, Qingdao, CHN; 4 Department of Metabolic Liver Disease, Qingdao Sixth People's Hospital, Qingdao, CHN

**Keywords:** zoonosis, food-borne parasitic disease, liver fluke, clonorchis sinensis

## Abstract

Clonorchiasis, an important foodborne parasitic disease, is prevalent in several Asian countries. In China, the three provinces with the highest incidence are Guangdong, Guangxi, and Heilongjiang, with no reported cases in Qingdao for nearly a decade. In this study, a 29-year-old male patient was diagnosed with fatty liver due to abnormal liver function during physical examination and was admitted to the hospital multiple times for examination and treatment within 3 years, but his liver function did not improve. Eventually, clonorchis eggs were found in the stool, confirming the diagnosis of clonorchiasis. The purpose of this report is to enhance the understanding of clonorchiasis among clinicians in no-prevalence areas, to familiarize laboratory technicians with egg identification, to strengthen parasite-knowledge training, and to reduce missed and misdiagnosed cases.

## Introduction

Clonorchiasis, also known as liver fluke, is caused by the consumption of raw or semi-raw freshwater fish and shrimp containing cysticercus. Adult worms parasitize the liver and bile ducts of humans (or cats and dogs), leading to a series of disease symptoms. This zoonotic parasitic disease seriously harms human health [1]. Individuals with early or mild disease often exhibit no obvious symptoms; consequently, the World Health Organization (WHO) lists liver fluke as one of the most neglected foodborne diseases globally [2]. Upon ingestion by humans, unripe freshwater fish or shrimp containing cysticercoids are unencapsulated in the duodenum and reach the intrahepatic bile duct within 10 to 20 minutes, following the bile’s flow through the common bile duct. Here they develop into adults and lay eggs with a lifespan of 20 to 30 years [1,3]. In cases of mild infection, there may be no clinical symptoms or subtle signs, making it difficult to detect. The primary detriment of clonorchiasis is liver injury. The lesions primarily manifest in the secondary liver bile duct. Adult worms inflict damage on the bile duct epithelium and submucosal blood vessels within the hepatic bile duct, as well as secretions, metabolites, and mechanical stimuli during parasite residence in the bile duct. Hypersensitivity and inflammatory reactions of the bile duct intima and peribile duct, bile duct epithelial hyperplasia, and bile duct constriction may result from mechanical stimulation and other factors. Sexual dilatation of the bile duct is typically cylindrical or sac-shaped. The majority of affected bile ducts display adenomatous changes, which may occur around the portal area when the infection is severe. Fibrous tissue proliferation and atrophic degeneration of liver cells, potentially progressing to the formation of biliary cirrhosis, ultimately contribute to liver cancer.

The WHO has declared liver fluke to be an important biological carcinogenic factor [4-6]. The disease is prevalent in several Asian countries, including China, South Korea, Vietnam, Thailand, and the Russian Far East, among others. In these regions, liver fluke continues to be a significant public health issue, with approximately 200 million individuals at risk of infection. Liver fluke has been prevalent in China for at least 2,300 years, and the number of infected individuals in the country is relatively high, with approximately 13 million people affected [7-10]. According to the second and third national surveys of major human parasitic diseases, the three provinces with the highest liver fluke infection rates were Guangdong, Guangxi, and Heilongjiang [11,12]. This case is the first reported case in the Qingdao area in the past decade. We comprehensively analyzed the clinical features, laboratory findings, imaging results, treatment, and prognosis of this case, thereby enhancing the awareness of healthcare professionals in non-endemic regions regarding clonorchiasis. Case details are provided as follows.

## Case presentation

Three years ago, a patient was found to have abnormal transaminase during a physical examination. Aside from minor tapping pain in the liver area, he displayed no other clinical symptoms or signs. The patient has been diagnosed with fatty liver multiple times by B-scan ultrasonography at other hospitals, but transaminase checks have revealed persistent abnormalities. In search of further diagnosis and treatment, the patient arrived at Qingdao Infectious Disease Hospital and was admitted to the outpatient department with a diagnosis of “liver dysfunction complicated by chronic hepatitis.” The patient’s family had no history of specific or infectious diseases. He did not have a habit of consuming raw or semi-raw foods. Post-admission, the potential liver function abnormalities induced by viral hepatitis, autoimmune hepatitis, medication, and alcohol consumption were ruled out.

The complete blood cell analysis revealed no significant abnormalities (Table 1).

**Table 1:** The results of a complete blood cell analysis

Tests	Results	Reference range
Haemoglobin	173g/L	120-160g/L
Red blood cell	5.47x10^12^/L	4-5.5x10^12^/L
Hematocrit	46.9%	40-54%
Mean corpuscular volume	85.70fl	86-100fl
Mean erythrocyte hemoglobin content	31.50pg	26-31pg
Mean corpuscular-hemoglobin concentration	369g/L	310-370g/L
White blood cell	8.78x10^9^/L	4-10x10^9^/L
Neutrophils	4.40x10^9^/L	2-7.7x10^9^/L
Lymphocyte	3.33x10^9^/L	0.8-4x10^9^/L
Monocyte	0.52x10^9^/L	0.12-0.8x10^9^/L
Eosinophil	0.49x10^9^/L	0.05-0.5x10^9^/L
Basophil	0.04x10^9^/L	0-0.1x10^9^/L
Platelet count	256x10^9^/L	100-300x10^9^/L

The biochemical report outcomes indicate the levels of alanine aminotransferase, glutamic oxalacetic transaminase, glutamine transpeptidase, triglyceride, total cholesterol, low-density lipoprotein, cholinesterase, and apolipoprotein B were all elevated, whereas the others were within the normal range (Table 2).

**Table 2:** Results of biochemical analysis

Tests	Results	Reference range
Total bilirubin	14.7μmol/L	3.4-20.5μmol/L
Direct bilirubin	5.3μmol/L	0-8.6μmol/L
Indirect bilirubin	9.4μmol/L	0-20μmol/L
Alanine aminotransferase	135U/L	0-55U/L
Glutamic oxalacetic transaminase	45U/L	5-34U/L
Alkaline phosphatase	71U/L	40-150U/L
Glutamine transpeptidase	83U/L	9-36U/L
Total protein	77.0g/L	64-83g/L
Albumin	48.4g/L	32-52g/L
Globulin	28.6g/L	20-45g/L
Albumin/Globulin	1.7	1.2-2.5
Prealbumin	365.8mg/L	220-400mg/L
Total bile acid	6.0μmol/L	0-10μmol/L
Triglyceride	2.32mmol/L	0-1.7mmol/L
Total cholesterol	5.80mmol/L	2-5.18mmol/L
High-density lipoprotein	0.91mmol/L	1.04-1.94mmol/L
Low-density lipoprotein	4.12mmol/L	2.07-3.10mmol/L
Cholinesterase	14219U/L	5000-12000U/L
Lipoprotein a	33mg/L	0-300mg/L
Apolipoprotein A	1.03g/L	1-1.6g/L
Apolipoprotein B	1.31g/L	0.6-1.1g/L
a -L-fucosidase	10.7U/L	0-40U/L

The color Doppler ultrasound revealed the gallbladder wall exhibited a thick and rough texture, with the parenchymal echo patch marginally thickened, enhanced, and dense. Additionally, the posterior echo demonstrated a weakened pattern. The patient’s stool was consecutively analyzed three times using an automated analyzer, and the outcomes were found to be normal.

**Figure 1:**
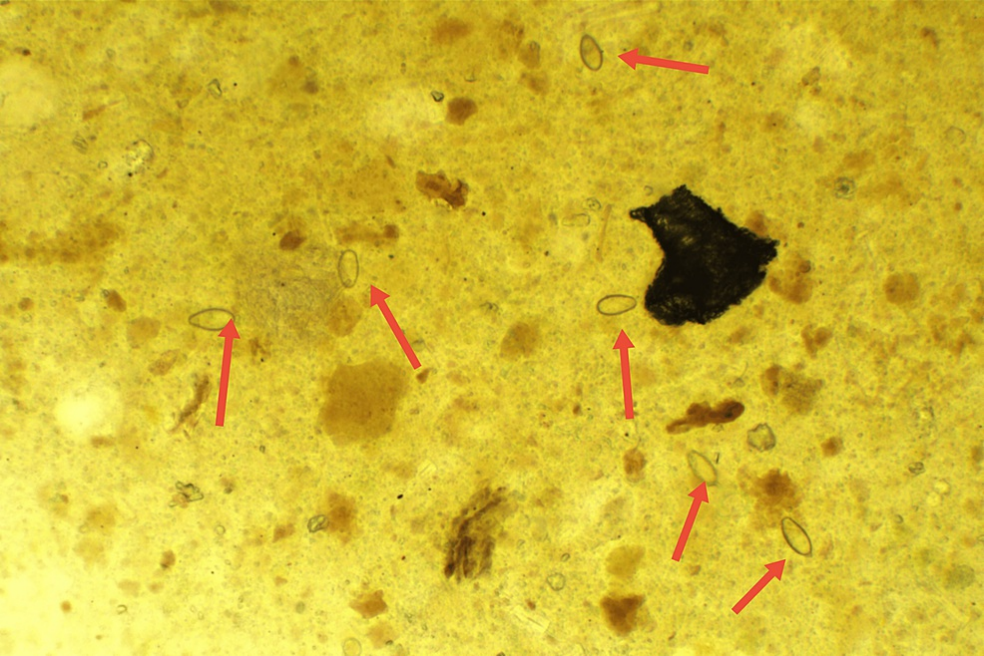
[×100] The red arrows denote the liver fluke eggs, which possess a shape akin to sesame seeds and measure approximately 26-32 μm × 15-17 μm in dimensions.

**Figure 2:**
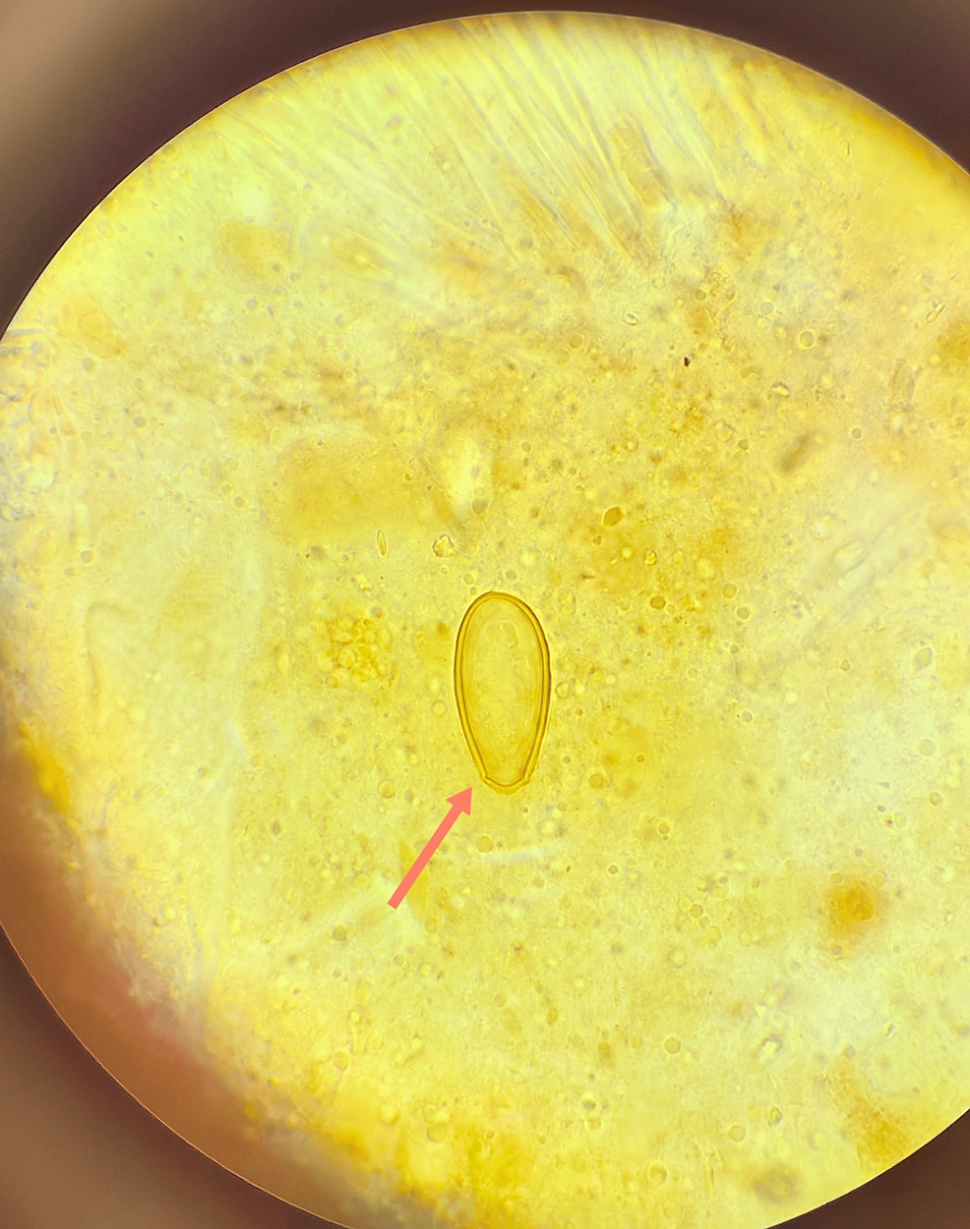
[×400] The red arrow signifies that the constricted end of the liver fluke egg serves as the egg cap, with the protrusions flanking the egg cap being the acromion.

**Figure 3:**
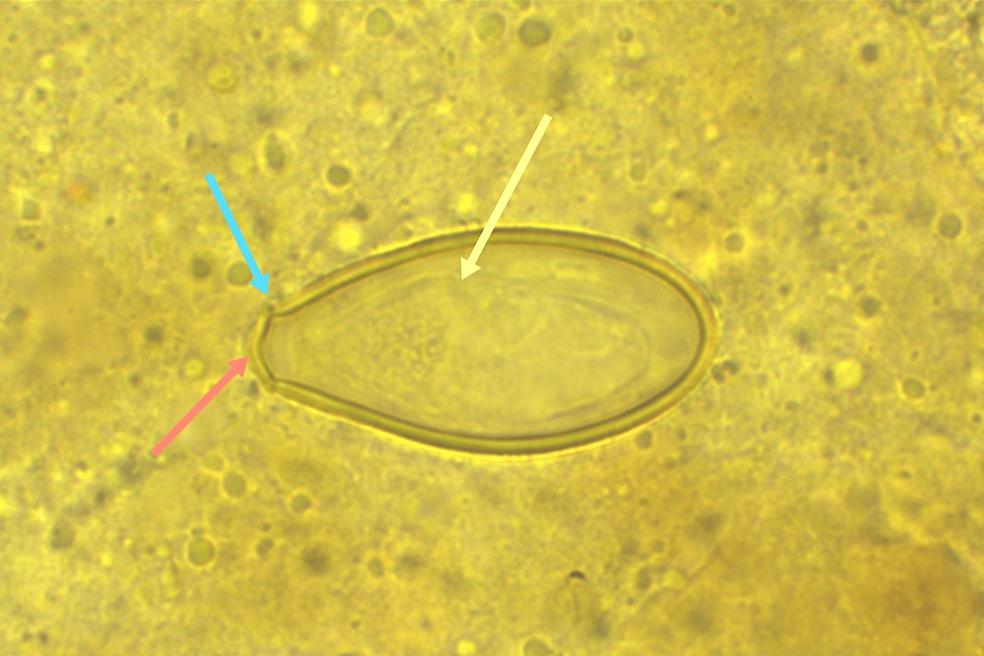
[×1000] The red arrow signifies the egg cap, the blue arrow denotes the acromion, and the yellow arrow indicates the content of liver fluke eggs, which is the larva.

**Figure 4:**
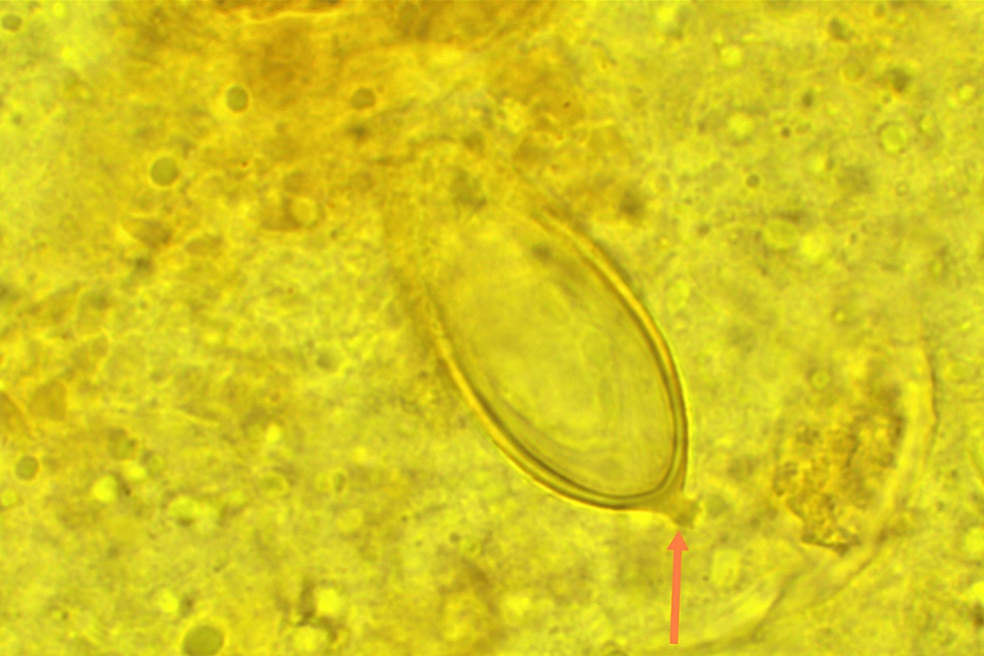
[×1000] In this illustration, it can be observed that the narrow end of the liver fluke egg is encompassed by fecal debris, whereas the broader end features a distinct, small cylindrical protrusion (illustrated by the red arrow).

Considering the patient’s medical history and physical characteristics and the elevated aminotransferase levels resulting from fatty liver a silybin capsule was administered as treatment. Subsequent liver function tests revealed no significant decline in transaminase levels. To rule out potential liver damage caused by parasites or bacteria, clinicians recommended that laboratory technicians meticulously examine the patient’s stool. For the fourth fecal examination, we employed the modified Kato thick smear technique - the application of the modified Kato thick smear method is appropriate for the detection of vermicular eggs in feces. This method involves covering the stool specimen with a 100-mesh nylon net, scraping approximately 50 mg of stool onto the net with a scraper, and then transferring it to the slide. Subsequently, the stool is covered with cellophane soaked in a glycerol malachite green solution, gently pressed, and spread out to an area of approximately 20mm×25mm. This step is followed by incubation in a 30-36 ℃ temperature box for approximately 0.5 hours or at 25℃ for about 1 hour. Once the stool film has slightly dried, it can be examined under the microscope. Finally, we detected liver fluke eggs under the microscope (Figures 1-4).

The patient was administered oral albendazole at 400 mg twice daily for a consecutive period of 7 days. When the patient was reexamined, a significant reduction in transaminase levels was observed, and no liver fluke eggs were detected in the patient’s stool.

## Discussion

Inhabitants of Guangdong, Guangxi, and other provinces have a penchant for consuming raw fish as well as drinking raw fish porridge. As well, individuals from Heilongjiang, Jilin, and other northeastern regions prefer to pair their raw fish with wine. This dietary habit is deemed as the primary factor contributing to the prevalence of liver fluke in these areas. According to the findings of the Third National Parasitic Disease Survey, the highest liver fluke infection rates were recorded at 6.68% in Guangxi, 1.91% in Guangdong, and 1.62% in Heilongjiang [12]. In contrast, the same survey in Qingdao demonstrated the absence of liver fluke infection [13].

As the catering, cold chain transportation, and tourism industries continue to thrive, the fusion of people’s culinary cultures has become increasingly widespread. This phenomenon allows individuals to sample dishes from around the world without even leaving their homes, thereby facilitating the dissemination of foodborne parasitic diseases in non-endemic regions. Liver flukes are easily misdiagnosed as hepatitis, cholecystitis, gallstones, and other ailments, particularly in non-endemic areas. This is primarily attributed to the limited experience of clinicians with these diseases and the unfamiliarity of laboratory technicians with worm eggs, which leads to misdiagnoses [14].

In the case report, the patient was born in Heilongjiang Province, pursued his studies in Guangxi Province for 3 years, and later relocated to Qingdao for employment. His dietary habits primarily consisted of commercially available food. It is postulated that there are three possible factors contributing to the patient’s liver fluke infection. First, the patient’s hometown is situated within an endemic area, thereby raising the likelihood of infection. Second, the patient resided and studied in Guangxi Province for an extended period, increasing the probability of an infection during his stay in this endemic region. Third, although Qingdao is a non-endemic area, the patient has consistently consumed takeaway food since starting work. The ingredients of these takeaway dishes are sourced from various regions across the country. If the food preparation is insufficiently thorough, the disease may still spread, thereby rendering it plausible that the patient contracted the infection post-employment. 

The patient’s liver function displayed repeated abnormalities over the course of three years, which was diagnosed as fatty liver. An analysis of the underlying cause was conducted. The Qingdao region is a non-endemic area, and local medical professionals lack familiarity with the knowledge and experience required to diagnose such conditions. Therefore, we urge disease control departments and medical institutions across all levels to intensify publicity and training efforts for foodborne parasitic diseases. Clinicians should pay attention to the identification of this aspect of the disease. For difficult cases, laboratory technicians must master the skills of manual filmmaking and microscopic search for pathogens.

Currently, the diagnostic criteria for liver fluke encompass epidemiology, clinical symptoms, laboratory tests, and other auxiliary examinations. Laboratory tests primarily involve enzyme-linked immunosorbent assays and etiological tests. Although enzyme-linked immunosorbent assays are straightforward, they possess a high rate of false positives and are unable to differentiate between past and current infections. Consequently, the primary method of laboratory diagnosis is based on the identification of liver fluke eggs in the patient’s stool and duodenal fluid as well as the surgical discovery of adult worms or eggs. Because of the invasiveness of the latter two methods, noninvasive etiological examination methods are more commonly employed, primarily focusing on the detection of eggs in feces. This detection process primarily employs two methods: the modified Kato thick smear technique and the aldehyde ether centrifuge precipitation method [15].

The patient was admitted to the hospital following three consecutive stool routine examinations that failed to detect liver fluke eggs. Most laboratories use automated analyzers for stool testing. There are various brands of automatic stool analysis instruments, and some brands are not sensitive to eggs - especially liver fluke eggs - because they are relatively small and easy to miss when the density is low. We employed an improved Kato method to search for liver fluke eggs in the stool. The clinical value of an imaging examination is significant for liver fluke screening because it enables the detection of various abnormalities in ultrasound images. These may include alterations in the echo of the intrahepatic bile duct wall; an increase, thickening, and enhancement of the bile duct wall echo; a mild or diffuse dilatation of the intrahepatic bile duct; the presence of bile duct calculus or local calcification; and so on.

Although imaging serves as a valuable auxiliary tool for liver fluke screening, its specificity is limited [16]. Furthermore, eosinophil counts are typically elevated during the acute phase of liver fluke infection, but they may normalize in the chronic phase. In the case of this patient, imaging revealed thickening and roughness of the bile duct wall. With the escalation of global travel and food imports, the threat of infection in non-endemic regions is also on the rise. As such, it is imperative for clinicians across all countries and territories to enhance the differentiation diagnosis of this illness. Consequently, we advocate for liver fluke screening in any patient exhibiting clinical signs of biliary or liver disease on imaging to facilitate early detection or exclusion of such conditions and prevent delays. For instance, initialize liver fluke antibody screening. If the screening result is positive, further stool examination for eggs can be conducted to confirm the presence of a liver fluke infection.

According to the WHO, early-stage liver fluke infection can be effectively treated by administering 25 mg/kg of medication three times daily for two to three days or a single dosage of 40 mg/kg, which can achieve a cure rate of over 90%. The side effects of praziquantel may include abdominal discomfort, vomiting, and hydrocephalus. Albendazole is another option [17,18]. Because of the patient’s concerns about praziquantel’s side effects, his healthcare provider prescribed oral albendazole 400 mg twice daily for seven days, in conjunction with other liver-protective treatments. After two months the patient experienced a significant reduction in transaminase levels, and no liver fluke eggs were detected in his stool.

## Conclusions

In summary, the clinical manifestations and indicators of hepatic fluorosis are atypical, which often leads to misdiagnosis or overlooked diagnosis, exacerbating the disease and resulting in severe consequences. Liver flukes can be effectively treated during their initial stages. In highly endemic regions, liver fluke-related tests have been incorporated into routine physical examinations. However, in non-endemic areas, lack of expertise and experience among young clinical practitioners, coupled with the unfamiliarity of laboratory staff with eggs, limited professional training, and the small size and low density of liver fluke eggs, often leads to missed detections, delaying the diagnosis and potentially progressing to chronic hepatic fluorosis, cirrhosis, or even liver cancer. Therefore, this case report aims to enhance the clinical medical professionals’ awareness of and attention to foodborne parasitic diseases, improve the ability of laboratory technicians to identify eggs, avoid overreliance on instruments, and provide manual microscopy for suspicious patients. It is also recommended that relevant authorities at all levels should intensify training and publicity efforts to reduce missed detections and misdiagnoses and minimize the incidence of foodborne parasitic diseases.

## References

[1] Lai DH, Hong XK, Su BX (2016). Current status of Clonorchis sinensis and clonorchiasis in China. Trans R Soc Trop Med Hyg.

[2] Wang ZY, Gu SW, Chen XQ, Cai YJ (2018). Clonorchiasis misdiagnosed as hepatic tumor: a case report. J Clinical Hepatobiliary Diseases.

[3] Tang Z, Shang M, Chen T (2016). The immunological characteristics and probiotic function of recombinant Bacillus subtilis spore expressing Clonorchis sinensis cysteine protease. Parasit Vectors.

[4] Reddy AK, Chakrabarty M, Liu Y, Cohen SH, Maniar AH (2021). Case report: Clonorchis sinensis infection associated with eosinophilic pneumonia. Am J Trop Med Hyg.

[5] Tang ZL, Huang Y, Yu XB (2016). Current status and perspectives of Clonorchis sinensis and clonorchiasis: epidemiology, pathogenesis, omics, prevention and control. Infect Dis Poverty.

[6] Na BK, Pak JH, Hong SJ (2020). Clonorchis sinensis and clonorchiasis. Acta Trop.

[7] Fürst T, Keiser J, Utzinger J (2012). Global burden of human food-borne trematodiasis: a systematic review and meta-analysis. Lancet Infect Dis.

[8] Bouvard V, Baan R, Straif K (2009). A review of human carcinogens--Part B: biological agents. Lancet Oncol.

[9] Qian MB, Yap P, Yang YC (2013). Accuracy of the Kato-Katz method and formalin-ether concentration technique for the diagnosis of Clonorchis sinensis, and implication for assessing drug efficacy. Parasit Vectors.

[10] Qian MB, Utzinger J, Keiser J (2016). Clonorchiasis. Lancet.

[11] (2005). A national survey on current status of the important parasitic diseases in human population. Chin J Parasitol Parasit Dis.

[12] Chen YD, Zhou CH, Zhu HH (2015). A study on the major human parasitic diseases in China. Parasitol China.

[13] Liu Suzhen, Ji Fengying, Li Xukui (2019). Human parasitic disease infection in Qingdao, Shandong Province. Chin Trop Med.

[14] Wang N, Tang B, Hao Y (2019). Acute shock caused by Clonorchis sinensis infection: a case report. BMC Infect Dis.

[15] Ying-Dan C, Ting-Jun Z, Long-Qi X, Bin Z, Yan-Hong X, Chang-Hai Z (2017). [Interpretation of diagnostic criteria for clonorchiasis]. Zhongguo Xue Xi Chong Bing Fang Zhi Za Zhi.

[16] Liang Zhi Cheng, Qiu Shu Zhong, Luo Li Xun (2015). Study on the correlation between liver and biliary B-ultrasound pathological changes and clonorchis sinensis infection. Chin J Schistosom Control.

[17] Xu LL, Jiang B, Duan JH (2014). Efficacy and safety of praziquantel, tribendimidine and mebendazole in patients with co-infection of Clonorchis sinensis and other helminths. PLoS Negl Trop Dis.

[18] Keiser J, Utzinger J (2010). The drugs we have and the drugs we need against major helminth infections. Adv Parasitol.

